# Systematic review and meta-analysis of the effect of increased vegetable and fruit consumption on body weight and energy intake

**DOI:** 10.1186/1471-2458-14-886

**Published:** 2014-08-28

**Authors:** Oliver T Mytton, Kelechi Nnoaham, Helen Eyles, Peter Scarborough, Cliona Ni Mhurchu

**Affiliations:** Centre for Diet and Activity Research, MRC Epidemiology Unit, University of Cambridge, Cambridge, UK; British Heart Foundation Health Promotion Research Group, Nuffield Department of Population Health, University of Oxford, Oxford, UK; Plymouth City Council, Armada Way, Plymouth, UK; National Institute for Health Innovation, School of Population Health, University of Auckland, Auckland, New Zealand

**Keywords:** Vegetable, Fruit, Body weight, Obesity, Energy intake, Trials, Adiposity, Nutrition

## Abstract

**Background:**

Increased vegetable and fruit consumption is encouraged to promote health, including the maintenance of a healthy body weight. Population health strategies (e.g. 5-A-Day or similar campaigns and subsidies on vegetables or fruit) that emphasize increased consumption may theoretically lead to increased energy intake and weight gain.

**Methods:**

We undertook a systematic review of trials that sought to increase vegetable and fruit consumption, in the absence of advice or specific encouragement to remove other foods from the diet, to understand the effect on body weight and energy intake. We included only randomised controlled trials. We pooled data using a random effects model for two outcomes: change in body weight and change in energy intake. Sensitivity and secondary analyses were also undertaken, including a one-study removed analysis and analysis by study sub-type to explore sources of heterogeneity.

**Results:**

A total of eight studies, including 1026 participants, were identified for inclusion in the review. The mean study duration was 14.7 weeks (range four to 52 weeks). The mean difference in vegetable and fruit consumption between arms was 133 g (range 50 g to 456 g). The mean change in body weight was 0.68 kg (95% CI: 0.15-1.20; n = 8; I^2^ for heterogeneity = 83%, p = 0.01) less in the “high vegetable and fruit” intake arms than in the “low vegetable and fruit intake” arms. There was no significant difference in measured change daily energy intake between the two arms (368 kJ; 95% CI: -27 to 762, comparing high vs low; n = 6; I^2^ = 42%, p = 0.07).

**Conclusion:**

Promoting increased fruit and vegetable consumption, in the absence of specific advice to decrease consumption of other foods, appears unlikely to lead to weight gain in the short-term and may have a role in weight maintenance or loss. Longer studies or other methods are needed to understand the long-term effects on weight maintenance and loss.

**Electronic supplementary material:**

The online version of this article (doi:10.1186/1471-2458-14-886) contains supplementary material, which is available to authorized users.

## Background

Regular consumption of vegetables and fruit protects against coronary heart disease, hypertension, stroke, some cancers and diabetes [1–4]. Many dietary guidelines emphasize the importance of a diet high in vegetables and fruit [5, 6]. Despite these guidelines, consumption of vegetables and fruit remains below recommended levels in many countries [7–9] and a substantial burden of disease globally is attributable to low consumption [10]. Consequently, strategies to increase fruit and vegetable consumption are a key focus for population health [11–13].

However, where such strategies focus on increasing vegetable and fruit consumption, without recommending substitution for other foods, there may be a risk that energy intake will increase, resulting in weight gain. For example, a randomised trial of subsidies on vegetables and fruit, found price discounts resulted in significantly increased purchases of vegetables and fruit with minimal change in the purchasing of other food items, which suggests that the total calories purchased increased [12]. Modelling studies, based on economic data, also suggest that subsidies on vegetables and fruit may result in an increase in calories purchased, as consumers can now afford more food [14, 15]. Moreover if all the additionally purchased calories were consumed the net effect on health, balancing the beneficial effects (e.g. reduction in cardiovascular disease) with the adverse consequences from weight gain may be neutral or even negative [14].

However, it remains unclear if increased purchases of vegetables and fruit, or increased availability, will result in increased energy intake and weight gain. Observational studies suggest that higher intake of vegetables and fruit is associated with lower body weight and reduced weight gain [1]. It has also been suggested that vegetables and fruit, in part due to their low energy density, may be more satiating in comparison with other foods of similar total energy content, [16, 17] which might lead to reduction in consumption of other energy dense foods from the diet. Consequently increased vegetable and fruit consumption is frequently encouraged to prevent weight gain and obesity [18].

Better evidence of whether increased availability of vegetables and fruits leads to weight change may come from trials. To date there has been one systematic review of the effect on adiposity from trials promoting vegetable and fruit intake, [18] and one meta-analysis of the effect of fruit and vegetable intake on body weight [19]. In the first review, among experimental studies in adults examining the effect of increased fruit or vegetable consumption, the majority (8/12) reported a reduction in body weight, although these positive studies were predominantly conducted in adults with a raised body mass index [18]. However, this review also included studies that changed other elements of the diet (e.g. swapping desserts for fruit), and so it is unclear if the observed changes were due to changes in vegetable and fruit consumption, or other components in the diet. No consideration was given to the significance of the observed effects and no formal meta-analysis was undertaken to quantify the effect size and measure its significance.

The second review concluded that studies to date do not support the proposition that recommendations to increase vegetables and/or fruit will cause weight loss, and suggests that these approaches are unwarranted as weight loss/maintenance strategies and that advice or measures to promote consumption should only happen alongside measures to reduce consumption of other food items (substitution) [19]. However in part due to stringent criteria, the primary analysis was based only on two studies.

Therefore, we set out to quantify the relationship between changes in vegetable and fruit intake, energy intake and body weight. We restricted our analysis to studies that attempted to promote or increase vegetable and fruit intake, without specifying other changes in the diet to closely replicate the scenario of increasing vegetable and fruit intake (e.g. by guidance or subsidies) in free living human populations. The PICO question was therefore defined as “what is the effect of an increased vegetable and fruit intake on either body weight or energy intake observed in randomised trials in free-living human populations compared to no increase in vegetable and fruit intake”.

## Methods

A full protocol was drawn up by one of the authors (OM). This was reviewed and approved by all other authors (copy of the protocol available as Additional file 1).

### Data sources and search strategy

A total of three databases were searched on the 3 September 2013: PubMed, Embase (Ovid) and Cochrane Trials database. The following search term was used in PubMed:

((((fruit) OR (vegetable [Title/Abstract])) AND ((energy OR calorie*[Title/Abstract]) OR (satiety [Title/Abstract]) OR (“body fat”[Title/Abstract]) OR (weight [Title/Abstract]) OR (“body mass index” OR BMI [Title/Abstract]))) AND (trial OR cohort OR observation* OR longitudinal [MeSH Terms])) AND “clinical trial”[Filter] AND “english”[Filter] AND “humans”[Filter]

In Cochrane the search term was: (Fruit or Vegetable) AND (Weight or BMI or “body mass index” or “energy” or “calorie*” or “body fat”), being restricted to trials only. In Embase (Ovid) the search term was (Fruit or Vegetable) AND (Weight or BMI or “body mass” or “energy” or “calorie*”), restricted to human studies, clinical tirals and English language paper. Only papers published in the last 25 years (i.e. on or after the 1 January 1988) were considered for inclusion.

### Study selection

We included randomised controlled trials (prospective studies with two or more arms, and participants being randomly allocated to the arms of the study). Eligible participants were adults or children. Studies were only included if they reported, or such measures could be derived from other reported measures, the difference in body weight or energy intake between the control and intervention adjusted for baseline measures. Where data for review outcomes were reported as measured but not included in the published manuscript, the authors were contacted directly to seek missing data.

The intervention had to promote or provide increased vegetables and/or fruit, and produce a different level of vegetable and fruit consumption, between at least two arms of the study to be included. We specified a minimum difference in fruit and vegetable consumption of 50 g or half a portion per day between arms. Interventions that sought to guide a switch from one food type to vegetables and fruit were excluded (e.g. switching from sweet deserts to fruit) as it would not be possible to identify whether any effect was due to changes in vegetables and fruit or other foods. Similarly interventions that sought to manipulate other dietary components simultaneously (i.e. a whole diet approach to weight loss) were also excluded, as the effect of changes in vegetable and fruit intake could not be isolated.

Vegetables and fruit included fresh, tinned or dried vegetables or fruit. Interventions based on purees (defined as a vegetable or fruit taken as a whole, with or without its peel, and put in a processor) were also included. Interventions based solely on vegetable or fruit juice were excluded, because of their association with weight gain, [20] and because fruit juices are less satiating than whole fruit [21]. Interventions involving a powder, extract or concentrate were also excluded.

Other exclusion criteria were: non-random allocation; inclusion of subjects with a medical illness that was liable to lead to weight loss or weight gain; studies that sought to manipulate energy intake to prevent or limit weight loss; the inclusion of other intervention components that might influence body weight (e.g. physical activity, or modification of other dietary factors) such that the effect of vegetable and fruit intake could not be isolated between the control and intervention arm; and studies in which the intervention period was less than two weeks.

### Data extraction

Data, including measures of study bias, were extracted using a standard data extraction sheet (copy included as Additional file 2) by two of the authors (OM and KN). The outcome measures extracted were change in mean energy intake (follow-up minus baseline) (measured in kJ) and change in mean body weight (follow-up minus baseline) (measured in kg), together with measures of variance. Where estimates by group, or the variance for the per-group change, were not reported, direct estimates of the mean difference (between control and intervention adjusted for baseline values) were used together with estimates of the variance. Where only a p-value was reported, we calculated the variance estimates from the provided data by using the z-score with the 2-tailed criteria [19].

All included studies were assessed for bias (at the study level) based on criteria used by the Cochrane Collaboration [22]. Criteria assessed were: selection bias (random allocation); performance bias (blinding of participants to weight gain); detection bias (blinding of observers); drop-out rates; source of funding (e.g. funded by vegetable or fruit producers); food provision (yes or no); setting (free-living or closed community); and measurement of diet (self-report or objective measure). As it was not possible to blind participants to the intervention, blinding of participants was not used as a criterion to discriminate between the studies.

### Statistical analysis

Meta-analysis was undertaken in Stata v11 (StataCorp. 2009. Stata Statistical Software: Release 11) using the “metan” command. The primary analysis compared the effect of intervention groups (“high fruit and vegetable intake”) with control groups (“low fruit and vegetable intake”) on two principal summary measures: difference in mean change in body weight (measured in kg) and difference in mean change in energy intake (measured in kJ). A random effects model was used, weighting the studies based on the standard error. A funnel plot was used to look for publication bias using the outcomes of change in body weight and change in energy intake. Heterogeneity was assessed with the I^2^ statistic.

We undertook sensitivity analyses around our primary analyses, using a ‘one-study removed’ analysis, whereby the meta-analysis is run multiple times each with a different and single study removed, was undertaken. This was to detect whether the observed effect was unduly influenced by any one study.

Secondary analyses were also undertaken. First, to test whether the observed effect was influenced by study design, two sub-types of study were identified: type a) interventions that encourage or support a general increase in fruit and/or vegetable consumption; and type b) interventions that provide a single vegetable or fruit for consumption with specific instruction about how much to consume. Meta-regression was undertaken to explore moderation by study type. Second, exploratory meta-regression analyses were undertaken to test for a dose–response relationship between vegetable and fruit intake and body weight or energy intake. Where information on consumption in grams was missing, it was assumed that one portion of vegetables of fruit was equivalent to 80 g.

## Results

**Figure 1:**
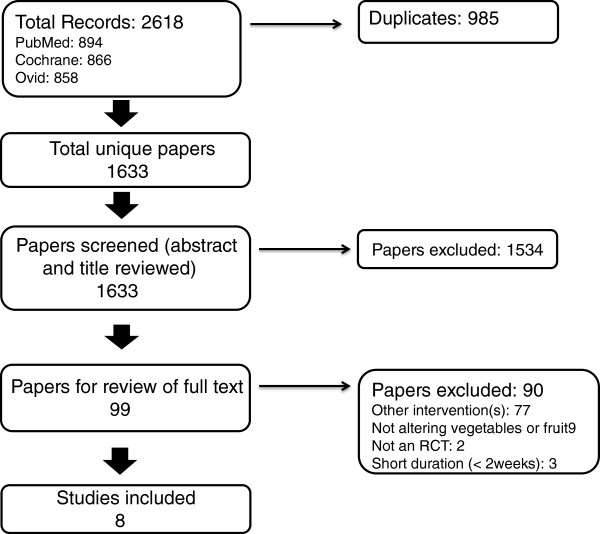
**Flow chart of papers identified, screened and reviewed.**

A total of 1633 unique papers were identified for possible inclusion in the study. After screening and exclusion of papers that did not meet the inclusion criteria, a total of eight studies, including 1026 participants, were identified for inclusion in the review (Figure 1). These eight studies provided nine sets of independent results with body weight as the outcome and six sets of independent results with energy intake as the outcome. Seven of these studies (eight arms) provided sufficient data for inclusion in a meta-analysis of effect on body weight, and five studies (six arms) provided sufficient data for inclusion in a meta-analysis of effect on energy intake.

### Description of studies

The eight studies are described in Table 1, with one study having two intervention arms [23]. All studies were randomised controlled trials, one of which had a cross-over design. All studies were conducted in adults, with no trials in children. The studies were predominantly conducted in North American (n = 5) or European (n = 2) populations (with one being conducted in India). Two studies explicitly recruited patients (type 2 diabetes diagnosed within past 12 months; patients with colorectal polyps). A further four studies recruited high risk adults (e.g. obese or high risk for cardiovascular disease). Consequently some groups of participants were obese. Only two studies recruited participants with a mean BMI below 25 kg/m^2^. The mean study duration was 14.7 weeks (range four to 52 weeks). The mean difference in vegetable and fruit consumption between arms was 133 g (range 50 g to 456 g).

**Table 1:** **Description of included studies**

	Type	Population	Mean body mass index (kg/m ^2^)	Study size (n)	Intervention	Intervention type	Daily difference in intake of fruit and vegetables between arms	Follow-up duration (weeks)
**Singh 1992** [26]	Parallel arm RCT	Adults with major risk factors for cardiovascular disease; India	24.3	463	Dietary advice focused on increasing fruit and vegetable intake	A	294 g	4
**Smith-Warner 2000** [27]	Parallel arm RCT	Patients with colorectal polyps; Minnesota, USA	27.7	201	Dietary advice focused on increasing fruit and vegetable intake	A	5.7 portions	52
**Whybrow 2007** [23]	Parallel arm RCT	Couples; Aberdeen, Scotland	23.7	62	Either 300 g or 600 g for fruit and vegetables provided daily	A	245 g and 433 g respectively	8
**Weerts 2009** [30]	Parallel arm RCT	Young overweight African American Women; USA	-	9	Gift card for fruit and vegetable purchases	A	1.2 cups per day	12
**Christensen 2013** [25]	Parallel arm RCT	Patients with newly diagnosed Type II Diabetes; Jutland, Denmark	32	63	Advice to eat at least two portions of fruit daily	A	184 g	12
**Basu 2010** [29]	Parallel arm RCT	Adults with metabolic syndrome; Oklahoma, USA	37.8	66	50 g blueberries provided daily	B	50 g*	8
**Peterson 2011** [28]	Cross-over RCT	Adults; California, USA	26.4	88	120 g figs provided	B	120 g*	10
**Dow 2012** [24]	Parallel arm RCT	Overweight and obese adults; Arizona, USA	32.1	74	Half a grapefruit provided for consumption with every meal	B	1.4 portions	6

*Assumed difference based on experimental design, actual difference not measured.

A variety of techniques were used to promote vegetable or fruit consumption. These included: simple dietary advice (n = 3); provision of free fruit and vegetables (n = 1); provision of a specific fruit with instructions about how much to consume (n = 3); and a store card allowing participants to purchase fruit and vegetables at no cost (n = 1). All interventions produced a difference in fruit and vegetable consumption between the control and intervention arm of at least 50 g/d. Four of the studies were classified as a type a intervention (promoting general consumption of vegetables and fruit), and four studies as type b intervention (promoting the consumption of one specific item of fruit). The duration of the intervention ranged from 4 weeks to 52 weeks.

### Study bias

Rating for bias of the eight included studies is shown in Table 2. No study was graded low on all criteria. Selection bias was frequently (n = 3) assessed as unclear as the methods of randomisation were often not described. Where randomisation was described studies were graded either low (n = 2) or medium (n = 2). However it appeared that participants were likely to have been blinded to the outcome of weight loss or change in energy intake as the primary aim of most studies was not to assess the effect on body weight. It was often unclear whether the assessors were blinded so most studies (n = 6) were graded as unclear for detection bias. Seven of the eight studies described drop-outs. Four studies had very high retention rates (above 90%) [24–27], two moderate (86%, 88/102, [28]; 73%, 58/66, [29]); and one low (69%, 62/90) [23]. With the exception of one study drop-outs were comparable between the different arms [27]. Two studies received funding from industry (typically in the form of provision of fruit from a fruit co-operative), although the design and conduct appeared to be independent of industry. A further three studies did not include information on funding so were graded as unclear. In all studies participants were free-living. In seven of the eight studies diet was assessed by questionnaire (e.g. food frequency questionnaire), with only one study having an objective measure of fruit and vegetable consumption (returned food).

**Table 2:** **Risk of bias in included studies**

	Selection	Performance	Detection	Drop-out	Funding	Food provision	Setting	Diet measurement
**Singh 1992** [26]	Unclear	Unclear	Unclear	Low	Unclear	High	High	High
**Smith-Warner 2000** [27]	Unclear	Medium	Unclear	Low	Low	High	High	High
**Whybrow 2007** [23]	High	Unclear	Unclear	Low	Low	Low	High	Low
**Weerts 2009** [30]	Medium	High	Unclear	High	Unclear	Medium	High	High
**Chistiensen 2013** [25]	Low	Unclear	Unclear	Low	Unclear	High	High	High
**Basu 2010** [29]	Unclear	Medium	Low	Low	High	Low	High	High
**Peterson 2011** [28]	Low	Medium	Unclear	Low	High	Low	High	High
**Dow 2012** [24]	Medium	High	Low	Low	Low	Low	High	High

Selection bias: low = both method of randomisation and concealment described; medium one of randomisation and concealment described; high = inadequate method of randomisation (e.g. order in which enrolled). Performance bias: low participants blinded to intervention and outcome of weight loss; medium = participants unaware of potential weight loss; high = participants not blinded to group or of potential for weight loss. Detection bias: low = assessors blinded to intervention; high assessors not blinded. Drop-out: low = drop-outs described; high = drop-outs not described. Funding: low = non-industry funding; high = funding by food or vegetable producers. Setting: low = closed living environment (e.g. institution); high = free-living individuals; food provision: low = vegetable or fruits provided; medium = vouchers to buy vegetable or fruits; high = participants advised to eat more fruit or vegetables, but have to purchase themselves. Diet measurement: low = observed or bio-markers measured; high = self-report.

### Primary analysis

**Figure 2:**
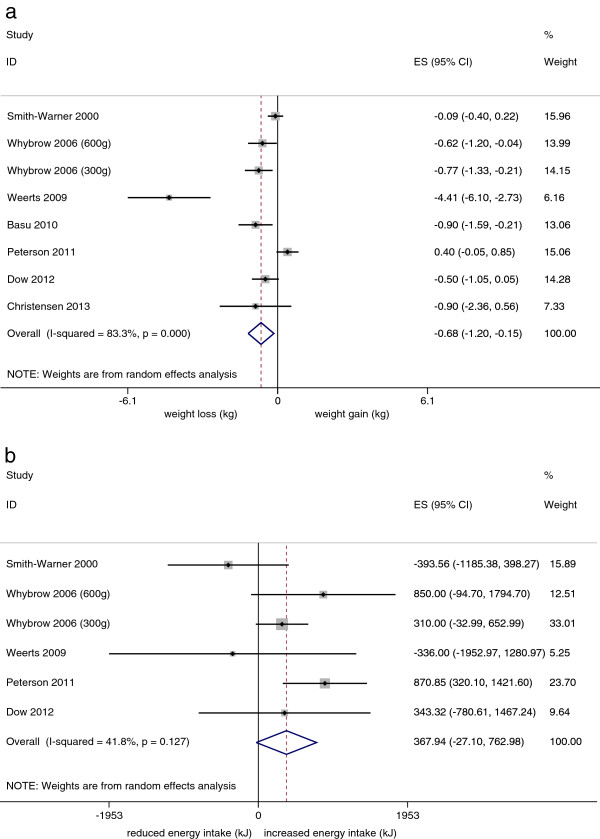
**Meta-analyses of the effect of high vegetable and fruit intake compared to low vegetable and fruit intake on body weight and energy intake. a**: Meta-analysis of the effect of high fruit and vegetable intake compared to low fruit and vegetable intake on change in body weight. **b**: Meta-analysis of the effect of high fruit and vegetable intake compared to low fruit and vegetable intake on energy intake.

The mean change in body weight was 0.68 kg (95% CI: 0.15-1.20; n = 8; I^2^ for heterogeneity = 83%, p = 0.01) less in the “high vegetable and fruit” intake arms than in the “low vegetable and fruit intake” arms (Figure 2a). There was no significant difference in measured daily energy intake between the two arms (368 kJ; 95% CI: -27 to 762, comparing high vs low; n = 6; I^2^ = 42%, p = 0.07) (Figure 2b).

### Sensitivity analysis

Undertaking a one study removed analysis did not change the direction or the significance of the finding with respect to body weight. The point estimates of effect size for body weight ranged from -0.39 kg to -0.85 kg comparing “high vegetable and fruit intake” to “low vegetable and fruit intake”. However one study, a small and outlying study, Weerts et al., [30] had a pronounced effect on the magnitude of the result. Removal of this study leads to an estimate of 0.39 kg (95% CI: 0.02-0.77; n = 7; I^2^ for heterogeneity = 68%, p = 0.04).

Undertaking the same analysis for outcome of change in energy intake, the point estimates of the change in energy intake ranged from 228 kJ to 489 kJ comparing “high vegetable and fruit intake” to “low vegetable and fruit intake”. Only with admission of one study, Smith-Warner et al., [27] did the result become significant.

**Figure 3:**
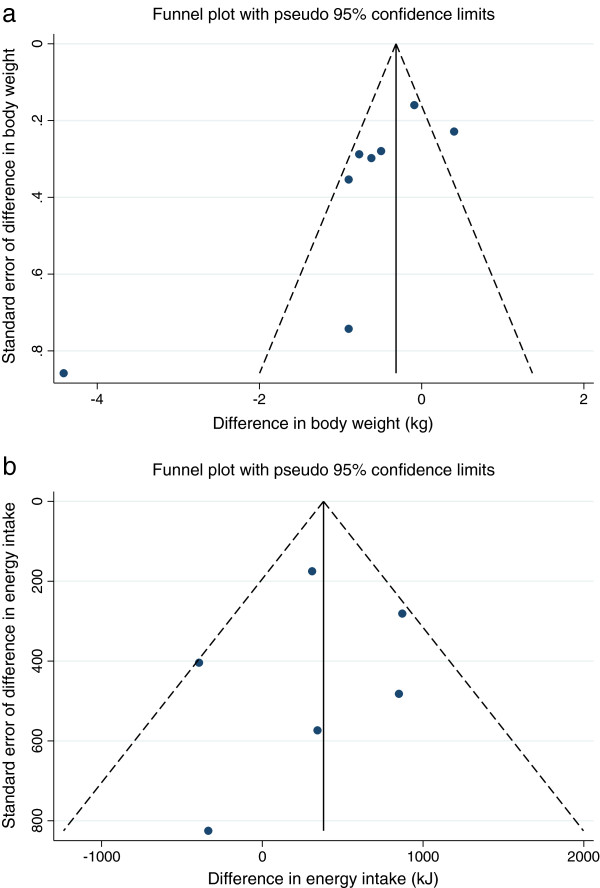
**Funnel plots for the outcomes of change in body weight and change in energy intake. a**: Funnel plot for the outcome of change in body weight. The funnel plot is a test for publication bias. Publication bias may be indictated by an absence of small negative trials, in this case trials missing from the bottom right hand corner. **b**: Funnel plot for the outcome of change in energy intake. The funnel plot is a test for publication bias. Publication bias may be indictated by an absence of small negative trials, in this case trials missing from the bottom right hand corner.

The funnel plot for the outcome of change in body weight is shown in Figure 3a and for the outcome of change in energy intake in Figure 3b. There was some evidence of asymmetry in the funnel plot for the outcome of change in body weight, which might suggest publication bias. However for both funnel plots the number of trials included was small limiting ability to make firm judgements about publication bias.

### Secondary analysis

There was some evidence of different effects for the two different types of intervention (type a: those that encouraged or supported a general increase in fruit and vegetable consumption vs type b: those that provided a specific fruit portion to be consumed on a daily basis). Change in body weight for type a studies (-1.03 kg, 95% CI: -1.84 to -0.22) was greater than for type b studies (-0.30, 95% CI: -1.08 to 0.48), although the differences were not significant on meta-regression (p = 0.21) and largely disappeared after elimination of the Weerts et al. study (-0.47 for type a studies vs -0.30 for type b studies).

For the outcome of change in energy intake, there was a smaller increase in energy intake among type a studies (change in energy intake: 193 kJ, 95% CI: -284 to 672) than for type b studies (change in energy intake: 768 kJ, 95% CI: 274 to 1263), although the differences were not significant (p = 0.93). We could not find strong evidence of a dose–response relationship between the difference in vegetable & fruit intake and change in body weight, on meta-regression (gradient = -0.210 kg per 100 g vegetable and fruit, p = 0.32)

## Discussion

### Statement of key findings

Our results show that trials of increased vegetable or fruit consumption, in the absence of guidance to reduce consumption of other foods, tends to result in either a small reduction in body weight or reduced weight gain relative to controls. The effect on energy intake is less clear. We did not find evidence of a dose response relationship between the increase in vegetable and fruit consumption and body weight.

### Limitations

The studies included in our meta-analysis were heterogeneous with respect to the intervention, design, study population, size and duration of follow-up. While all studies were trials, due to the nature of the design some studies were still at risk of bias. In particular all studies relied wholly or largely on standard questionnaires or recall to record diet intake (including energy intake) rather than objective measures. Given all interventions were focused on vegetable and fruit intake it is possible that the estimates of vegetable and fruit intake were biased (e.g. being over-estimated in the intervention group). Dietary surveys might also be a poor means (insufficiently sensitive) to detect the relatively small changes in energy intake sufficient to explain the observed changes in body weight, reflected in the wide confidence intervals. For these reasons and because body weight was objectively measured we place greater emphasis on the finding’s concerning body weight compared to energy intake.

None of our studies were double-blind. Practically it was not possible to blind subjects to the intervention. However the primary outcome of most studies was a change in markers of cardio-metabolic disease (e.g. blood pressure, glycaemic control, serum lipids) so many subjects may have been unaware of any potential impact on weight loss. The subjects were also all free-living, which may have resulted in less certainty about other changes in behaviour, but this was necessary to replicate the scenario of people being able to adjust and adapt other aspects of their diet, in real-life settings, in response to recommendations/guidance to increase vegetable and fruit consumption.

The meta-analysis is based on a small number of studies and a small total number of participants (n = 1026). Consequently our findings should be treated with a degree of caution. The meta-analysis does show significant heterogeneity, in other words the effects of different interventions are different. We have not been able to explain this based on our pre-specified hypothesis about the form of the interventions. However because of the limited dataset, our ability to explore causes of heterogeneity is limited. A variety of factors may explain the heterogeneity: duration of intervention, baseline body weight, interaction with other lifestyle factors (e.g. physical activity), type of vegetable or fruit (e.g. energy density, fibre content, carbohydrate type), and the nature of the dose–response relationship. While we cannot rule out publication bias as an explanation for our findings, we find no evidence of publication bias or small study bias in our funnel plots.

The study duration is typically short and the dose of fruit and vegetables relatively modest (mean of just over one and half portions per day). Consequently in some studies it may not have been physiologically likely that changes in body weight would have been observed. This might be particularly likely in individuals of low or normal body weight, in whom weight maintenance (rather than loss) is more likely (and desirable) goal. Consequently despite the absence of a noticeable effect on body weight, one should be cautious about concluding that vegetables and fruit do not have an important role in weight maintenance (or loss).

No studies were identified in children so one should be cautious about extrapolating the findings to children. While trials have been undertaken in children, [31] our review did not identify any trials in children, in part because the trials in children have been cluster randomised. Children might be better treated in a separate meta-analysis given that they are growing and so naturally experiencing an increase in body mass, for which it might be necessary to relax the criteria around individual-randomisation.

### Comparison with other studies

While our systematic review differs from previous reviews [18, 19] the headline findings, in terms of bodyweight, may appear comparable. In the first review the majority of trials identified reported that increased vegetables and fruit consumption was associated with weight loss (ranging from 0.8 kg to 7.9 kg; n = 8) with the remaining trials (n = 4) reporting no effect, [18] which might be consistent with an overall effect of small weight loss (or reduced weight gain). However the trials in this review manipulated other aspects of the diet, alongside vegetable and fruit intake and the observed effects might be attributable to other dietary changes. Only one trial [23] was shared between the two reviews.

The second review, also a meta-analysis, addresses the same question that we identified. However because of the nature of our search strategies and inclusion/exclusion criteria we have identified a largely different set of studies, again with only one over-lapping study. This study only considered the effect of vegetable and fruit intake on body weight and not on energy intake [23]. Excluding the outlier our point estimates are comparable (-0.39 kg vs -0.16 kg) with overlapping confidence intervals, although our small reduction is significant and in contrast the estimate from the Kaiser review is non-significant. Both reviews include a similar number of participants (1194 vs 1053).

Other evidence concerning the effect of vegetables and fruit on bodyweight, comes from observational and cohort studies. In these studies the findings may be influenced by other confounding factors (related to diet and other aspects of lifestyle). They may also reflect longer term influences (over a period of years) of vegetables and fruit on body weight. Cohort studies show either an inverse association between vegetable or fruit consumption and body weight and some studies showing a null result [32, 33]. Again this may be consistent with our finding of a small negative effect on body weight of greater vegetable and fruit consumption.

Our findings in terms of change in energy intake appear to contrast with the previous review, which suggested that a reduction in energy intake was associated with increased vegetable and fruit consumption [18]. By including studies that increased vegetables and fruit by making switches with other food items (e.g. replacing desserts with fruit), this earlier review will have included trials where energy intake may be more likely to fall and for that fall to be captured by the dietary record. In contrast, our review only included studies of free living individuals where ‘switching’ was not specifically promoted.

### Meanings of the study and mechanisms

Some factors in our analysis support the hypothesis that promotion of vegetable or fruit intake, in the absence of guidance on what to reduce in the diet, will lead to weight loss or prevent weight gain. Our primary analysis showed a small significant body weight reduction or reduced gain in body weight, over a short period of time. While the effect size appears small given the short mean study duration, over the longer-term the effect could be more clinically meaningful. The direction of effect persisted when we undertook sensitivity analyses (one study removed). On the other hand some factors do not support this hypothesis. The effect size is small. We did not find strong evidence of a dose–response relationship and we could not find evidence of a reduction in energy intake, although considerable uncertainty surrounds the estimate of the effect on energy intake.

We conclude that interventions that seek to generally promote increase in fruit and vegetable consumption (when not specifying what to reduce or substitute) are unlikely to lead to weight gain and could result in a small amount of weight loss. This is reassuring for interventions (e.g. “More Matters” and “5-A-Day” campaigns [11, 34] or subsidies on vegetables and fruit) designed simply to increase vegetable and fruit consumption in order to improve health. Even where such programmes fail to prevent obesity or cause weight loss, significant health benefits are likely to accrue from improvements in cardio-metabolic health and cancer risk [1].

Changes in energy intake are less clear. A finding of no change in energy intake or even a slight increase may appear at odds with the findings of reduced body weight. This may be an artefact of how diet is recorded, such that people may not report the small changes in frequency or portion size in other food items that may have occurred to compensate for the increased intake of vegetables and fruit. However it may be real. Energy intake is measured using combustible energy, but the fraction of energy that the body can absorb and metabolise from vegetables and fruit is much lower than for many other foods [35]. Therefore it is plausible that energy consumed could be constant or increase but that the energy absorbed by the body would fall.

Given the limited evidence base, further studies are warranted. Studies that increase the availability of vegetables and fruit generally (rather than specific items), and accurately record dietary substitution as well as body weight would be particularly informative. Further research should also consider the importance of vegetables and fruit in the long-term; the effects in overweight and normal weight, including their role weight maintenance; as well as considering the effect of directed substitution versus addition of vegetables and/or fruit.

## Conclusion

Promoting increased fruit and vegetable consumption (in the absence of specific advice to decrease consumption of other foods) in the short-term appears unlikely to lead to weight gain, and may have a role in weight loss or maintenance of a healthy weight.

## Electronic supplementary material

Additional file 1: **Protocol for systematic review.** (DOCX 28 KB)

Additional file 2: **Data Extraction Sheet.** (DOCX 18 KB)
