# Erratum to: systematic review and meta-analysis of the effect of increased vegetable and fruit consumption on body weight and energy intake

**DOI:** 10.1186/s12889-017-4664-2

**Published:** 2017-08-17

**Authors:** O. T. Mytton, K. Nnoaham, H. Eyles, P. Scarborough, C. Ni Mhurchu

**Affiliations:** Centre for Diet and Activity Research, MRC Epidemiology Unit, University of Cambridge, Cambridge, UK

## Erratum

There were some errors in the original published article [1]. Firstly relating to the calculation of the confidence intervals for four of the studies included in the meta-analysis, [2–5] which are detailed below. Secondly one of the papers, Whybrow et al. was incorrectly cited as being published in 2007, when it was published in 2006 [2].

Description of errors relating to derivation of 95% confidence intervals

The study by Whybrow et al. had three arms (control, arm A, arm B) [2]. We had effectively included the control arm twice by using it as the control for both arm A and arm B of the study. We have now halved the size of the control group (allocating eight participants in one control group and nine to the second control group), which is one approach to correct for the potential problem of double counting the control group [6].

We used an incorrect approach to derive standard errors for the difference in change between two arms of the study, from the standard errors for change in each arm of the study. This affected three studies [2, 5, 7]. We have now corrected this.

Third there was a transcription error made when extracting data from the paper by Christensen et al. [4]. The correct *p*-value for the difference in change in body weight between the control and intervention arm is 0.18.

Additional files 1 and 2: Tables S1 and S2 below show the derivation of the standard error for the difference in change in body weight between the control and intervention group, deriving standard errors from *p*-values and from standard errors for the change in body weight within each group respectively.

Effect of these errors on results

The error in derivation of standard errors effects Figs. 2a and 3a in the original manuscript. Revised copies of these figures are shown below.

**Fig. 2:**
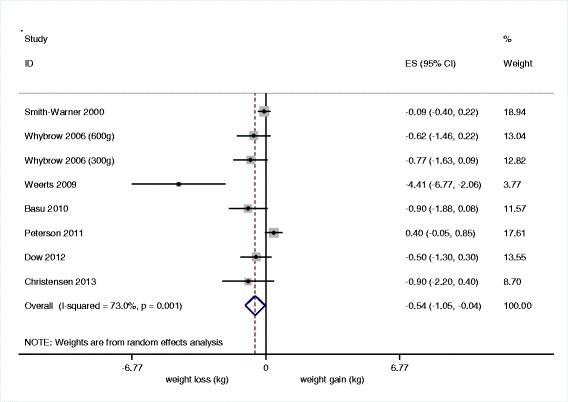
Revised figure 2 **a** Meta-analyses of the effect of high vegetable and fruit intake compared to low vegetable and fruit intake on body weight (Amended)

**Fig. 3:**
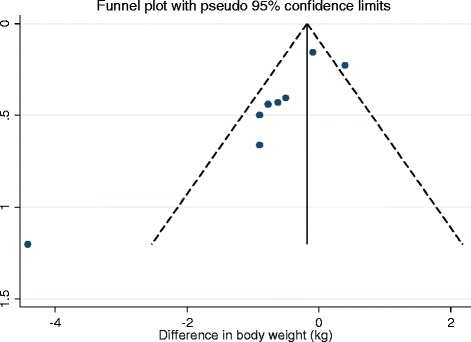
**a** Funnel plots for the outcomes of change in body weight **(**Amended)

The first line of the Results, under Primary Analysis, should read: “The mean change in body weight was 0.54 kg (95% CI: -1.05 to −0.04; *n* = 8; I^2^ for heterogeneity = 73%, *p* < 0.01) less in the ‘high vegetable and fruit’ intake arms than in the ‘low vegetable and fruit intake’ arms (Fig. 2a).”

The equivalent line in the Results section of the Abstract should read: “The mean change in body weight was -0.54kg (95% CI: -1.05 to -0.04; n=8; I^2^ for heterogeneity = 73%, p<0.01) less in the ‘high vegetable and fruit’ intake arms than in the ‘low vegetable and fruit intake’ arms.”

The first paragraph of the Sensitivity Analysis should be amended to read: “Undertaking a one study removed analysis did not change the direction of the finding with respect to body weight. The point estimates of effect size for body weight ranged from -0.32 kg to -0.74 kg comparing ‘high vegetable and fruit intake’ to ‘low vegetable and fruit intake’. The difference only remained significant when two of the seven studies, Petersen et al and Smith-Warner et al, were removed. After removal of Weerts et al, the effect estimate was -0.32 kg (95% CI: -0.71 to 0.06; n=7; I^2^ for heterogeneity = 56%, p=0.04).”

The second sentence of the Secondary Analysis section should read as follows: “Change in body weight for type a studies (-0.86 kg, 95% CI: -1.65 to -0.07) was greater than for type b studies (-0.25, 95% CI: -1.08 to 0.58), although the differences were not significant on meta-regression (p = 0.19) and largely disappeared after elimination of the Weerts et al. study (-0.38 for type a studies vs -0.25 for type b studies).” The last sentence of this section should read: “We could not find strong evidence of a dose–response relationship between the difference in vegetable & fruit intake and change in body weight, on meta-regression (gradient = -0.150 kg per 100 g vegetable and fruit, p = 0.32).”

The third sentence of the second paragraph under the section entitled “Comparison with other studies”, in the Discussion should read: “Excluding the outlier our point estimates are comparable (-0.32 kg vs -0.16 kg) with overlapping confidence intervals.”

Effect of these errors on conclusions

In summary the use of the amended standard errors has had two principle effects. First our estimate of the primary effect size is more conservative (−0.54 kg vs −0.68 kg), due to re-weighting of the studies. Although the observed decrease is still statistically significantly. Second when we remove the outlier the difference is no longer significantly different to zero. Removing other studies (individually) also results in a non-significant result.

While the revised results may provide weaker evidence to support an assertion that increases in fruit and vegetable consumption may result in loss of body weight, we think the overall effect of these statistical errors on our results and conclusions is slight.

In our manuscript, our conclusion was intentionally worded cautiously, reflecting greater uncertainty than was captured in the published confidence intervals (e.g. due study quality and study design). Our original conclusion was that “Promoting increased fruit and vegetable consumption, in the absence of specific advice to decrease consumption of other foods, appears unlikely to lead to weight gain in the short-term and may have a role in weight maintenance or loss.”

We do not feel the amended results alter these conclusions. Whilst some of the results could (e.g. the estimate after removal of the Weert’s et al. study) be consistent with a gain in body weight, the amended results appear most consistent with an increase in fruit and vegetable consumption having either no effect on body weight, or a small reduction.

## Additional files

Additional file 1: Table S1. Derivation of standard errors for the difference in change in body weight between control and intervention from *p*-values (necessary for four studies included in the review). T-score and standard errors were imputed in Microsoft Excel, following the process outlined in the Cochrane Handbook (section 7.7.3.3) [8]. (DOCX 12 kb)

Additional file 2: Table S2.Derivation of standard errors for the difference in change in body weight between control and intervention from standard errors for each group (necessary for three studies). *size of control group halved; and estimate of SE correspondingly adjusted (values used in calculations are shown in the table); the following formula used to calculate the standard errors for the difference in change in body weight between control and intervention was: ${SE}_{\mathit{\operatorname{int}}- con}=\sqrt{\frac{var_{int}}{n_{int}}+\frac{var_{con}}{n_{con}}}$… (DOCX 12 kb)
