# Supplementary material for: Systematic review and meta-analysis of the effect of increased vegetable and fruit consumption on body weight and energy intake
Source: BMC Public Health. 2014 Aug 28;14:886. doi: 10.1186/1471-2458-14-886 (PMC4158137; doi:10.1186/1471-2458-14-886)
Supplement: Supplementary file 1 — Additional file 1: Protocol for systematic review. (DOCX 28 KB) [file 12889_2014_7014_MOESM1_ESM.docx]

**Protocol:**

**Systematic review of effect of changes in fruit and vegetable consumption on weight**

**Background**

Consuming fruit and vegetables is beneficial for health. Subsidies or other means to reduce the price of fruit and vegetables has been proposed as one means to encourage consumption. Economic data shows that consumption of fruit and vegetables increases as the price falls. However even allowing for changes in consumption of other foods this economic data suggests that more calories are purchased as the price of fruit and vegetables falls. Health modelling suggests that the net effect of these purchasing changes (assuming these reflect changes in consumption) would be to increase weight (from the increase in energy intake) that would offset the direct beneficial effects of fruit and vegetable consumption.

This runs counter to longitudinal data on food prices and health, which suggest that as fruit and vegetable prices fall, consumption rises and body weight falls. Furthermore it has been suggested that fruit and vegetables may promote weight loss via effects on satiety; and the fibre content within fruit and vegetables may reduce the absorption of energy. A systematic review of the effect of fruit and vegetable intake (LeDoux, 2011) concluded that it was unclear whether changes in fruit and vegetable intake influenced body adiposity (including body weight). This systematic review did not formally quantify the relationship in a meta-analysis. Instead it expressed the relationship as the total number of positive, null and negative studies.

This review aims to quantify this relationship, undertake sensitivity analysis considering the strength of the evidence, and test the relative importance of fruit compared to vegetables. Quantifying the relationship may provide a more accurate means to address the question. It will also be important for developing a more refined way to model the effect of food price changes on health, by disaggregating the effects of changes in the composition of the purchased diet from the energy intake of the purchased diet.

**Aim**

To quantify the relationship between total fruit & vegetable intake and body weight and/or energy balance, in adults and children

**Methods:** Systematic review and meta-analysis of randomised controlled trials

**Inclusion Criteria:**

Study type: Randomised controlled trials

- Prospective study
- Two or more arms
- Participants randomly allocated to the arms of the study

Subjects:

- Humans (either adults or children)

Intervention type:

- Any intervention that produces a different level of fruit and/or vegetable consumption between at least two arms of the study

Fruit and vegetables would include:

- Fresh fruit and vegetables
- Dried fruit and vegetables
- Purees (Puree is when a vegetable or fruit is taken whole and put in processor and the whole fruit/vegetable is processed (+/- peel))

Fruit or vegetable juices would be excluded

Outcomes - any of the following:

- Body weight
- Body mass index
- Energy Intake
- Percentage body fat
- Fat mass
- Waist circumference
- Any other measure of body fatness or body weight

**Exclusion criteria:**

- Non-random allocation
- Subjects with a medical illness that may lead to weight loss, or weight gain
- Any intervention where other factors (e.g. other dietary components) or physical activity differ between the two arms of the study
- Less than two weeks duration

**Search Strategy**

Data sources: Medline, Embase and Cochrane Central Registrar of Controlled Trials

Pubmed (5 Feb 2013): 843

((((fruit) OR (vegetable[Title/Abstract])) AND ((energy OR calorie*[Title/Abstract]) OR (satiety[Title/Abstract]) OR ("body fat"[Title/Abstract]) OR (weight[Title/Abstract]) OR ("body mass index" OR BMI[Title/Abstract]))) AND (trial OR cohort OR observation* OR longitudinal[MeSH Terms])) AND "clinical trial"[Filter] AND "english"[Filter] AND "humans"[Filter]

Cochrane (5 February 2013 ):866

(Fruit or Vegetable)

AND (Weight or BMI or "body mass index" or "energy" or "calorie*” or “body fat”)

Restricted to Trials

Embase (Ovid) (15 Feb 2013): 820

Search terms (as keyword)

(Fruit or Vegetable)

AND (Weight or BMI or "body mass" or "energy" or "calorie*”)

Restricted to: Human studies, clinical trial , English language; from 1988 only

**Data collection and analysis**

Four researchers (OM, PS, CNM, HE) will review titles and abstracts to identify potentially relevant studies, using the selection criteria for eligible study designs, participants, and interventions. Studies that clearly fail to meet these inclusion criteria will be excluded. Those that meet these criteria or that cannot be definitely excluded by both researchers will be obtained in full text to exclude those that do not meet the inclusion criteria (see notes in appendix).

Two researchers (OM and KN) will then review the full text of the identified papers to determine eligibility. If eligbility is unclear after full-text review, authors will be contacted for clarification. If the two researchers are unable to agree about eligibility after discussion, a third reviewer (PS) will review the full text to determine eligibility.

**Data extraction and management**

Data will be extracted by one of two researchers (OM and KN) from all documents eligible for this review using standardized paper forms. Data extraction will consist of information on study design, intervention type and characteristics, participants, setting, methods, outcomes, and results. Authors will be contacted to obtain missing information. Data will be entered by one investigator (OM) into Microsoft excel for analysis.

**Assessment of risk of bias in included studies**

Two review authors (OM and KN) will independently assess the risk of bias based on criteria used by the Cochrane Collaboration, as set out in their handbook. A judgement against each criteria for risk of bias will be made as follows:

- Low risk of bias
- Medium risk of bias (NB – not always used)
- High risk of bias
- Uncertain

**Quality Criteria**

Selection bias (Random allocation)

- Low risk = Describe method of randomisation and describe concealment of allocation
- Medium risk = describes EITHER method of randomisation or concealment of allocation
- Uncertain = methods of randomisation not described
- High risk = not adequately randomised (likely not included in study)

Performance bias

- Low risk: Participants blinded (in practice likely that all studies will fail on this account) to the group AND the potential outcome of weight loss
- Medium risk participants blinded to outcome of weight loss
- High risk: participants not blinded to allocation group and not blinded to possibility that fruit and vegetables may lead to weight loss

Detection bias (report for each outcome measure)

- low risk = describe all measures used, if any, to blind outcome assessors from knowledge of which intervention a participant received

Drop-out

- Low risk: describes drop-out rate
- High risk: drop out is not described

Other sources of bias: funding

- Low risk = not funded by industry (i.e. fruit and vegetable producers, or related industries)

Other sources of bias: setting

- low risk of bias = closed study
- high risk of bias = open (free-living individuals)

Other sources of bias: type of intervention

- low risk of bias = provision of food
- high risk of bias = dietary advice and participants to all buy their own food

Other sources of bias: measurement of diet

- low risk of bias = self-report (please note method)
- high risk of bias = observed or biomarkers to validate

**Analysis of data**

The primary outcome will be body weight (this is considered at lower risk of bias than energy intake, which is self-reported). Secondary outcome measures will be energy intake and other measures of adiposity (as permitted by the data extracted).

A forest plot will be undertaken to assess the likelihood of publication bias.

Analysis will not be on intention to treat, as our primary question is concerning efficacy (i.e. what is the effect of fruit and vegetables on energy balance and body weight, and not the effectiveness of interventions).

We will examine study characteristics in term of participants, interventions (within broadly categorized types of interventions – see Data Synthesis below) and outcomes for evidence of clinical heterogeneity. Statistical heterogeneity will be analysed by the Chi^2^ test and I^2^ statistic. The I^2^ statistic describes the percentage of total variation across studies attributed to heterogeneity rather than by chance (Higgins, 2003). Because meta-analyses generally have very few studies, detection of heterogeneity is not very precise due to low power; therefore, a P value of 0.10 will be our set alpha (Dickersan, 1992).  We will accept the studies as heterogeneous if the I^2^ statistic is greater than or equal to 50% with a P value less than 0.10.

**Subgroup analysis and investigation of heterogeneity**

Depending on the availability of data, we will explore subgroup heterogeneity for primary outcomes by using random effects modelling as described by Borenstein (Bronstein 2009), on the following characteristics:

1. Fruit vs Vegetable
2. Duration of intervention
3. Gender
4. Age group
5. Disease condition vs healthy

**Sensitivity analysis**

We will perform sensitivity analyses to determine the influence on primary outcomes on each of the reported indices of quality and overall pre-assigned cut-point for quality (randomised, blinded to outcome of weight loss, provided food and/or objective means to measure actual diet consumed, not industry funded)
