# Supplementary material for: Systematic review and meta-analysis of the effect of increased vegetable and fruit consumption on body weight and energy intake
Source: BMC Public Health. 2014 Aug 28;14:886. doi: 10.1186/1471-2458-14-886 (PMC4158137; doi:10.1186/1471-2458-14-886)
Supplement: Supplementary file 2 — Additional file 2: Data Extraction Sheet. (DOCX 18 KB) [file 12889_2014_7014_MOESM2_ESM.docx]

**Data Extraction Sheet: Fruit and Vegetable intake, energy intake and body weight meta-analysis**

| Reviewer initials | Publication Year | | Journal |
| --- | --- | --- | --- |
| Brief title and/or author | | | |
| Are the inclusion and exclusion criteria met? | | Yes/ No | |

| **Other notes on participants and study** | | | | |
| --- | --- | --- | --- | --- |
| Who are the participants | |  | | |
| Where was the study conducted (town/country)? | |  | | |
| Was the intervention explained to the study participants as a weight loss intervention | | | Yes No Unclear | |
| **Risk of bias (see notes in protocol)** | | | | |
| Quality indices | **Notes on** | | | **Overall judgement**  **(low/medium/high/uncertain)** |
| Selection bias |  | | |  |
| Performance bias |  | | |  |
| Detection bias |  | | |  |
| Drop-out |  | | |  |
| Funding |  | | |  |
| Setting |  | | |  |
| Food provision |  | | |  |
| Diet measurement |  | | |  |
| **Other** | | | | |
| Any other papers identified that should be considered for inclusion? If so give author name, journal and year | | | | |
| Other comments: | | | | |

*Please circle to indicate which measure is given

|  | **Arm A** | **Arm B** | **Arm C** |
| --- | --- | --- | --- |
| Nature of intervention  And how implemented |  |  |  |
| Number who started study  If given add (females/males) |  |  |  |
| Number who completed study  If given add (females/males) |  |  |  |
| Mean or Median* age or participants |  |  |  |
| Range/SD/SE* of age of participants |  |  |  |
| Quantity of fruit and vegetables consumed (add units)  If given split (fruit/vegetables) |  |  |  |
| Range/SD/SE* of FV consumption |  |  |  |
| Total energy intake at baseline (add units) |  |  |  |
| Range/SD/SE* of energy intake at baseline |  |  |  |
| BMI of participants  (With range/SD/SE)* |  |  |  |
| Other measure of body fatness if given, add units and add measure for range/SD/SE |  |  |  |
| Duration of study  (add units) |  |  |  |
| **Outcomes (please report on those who completed study not on intention to treat);** if only intention to treat is given please add note to state this | | | |
| Change in body weight (add units) |  |  |  |
| Range/SD/SE* for change in body weight |  |  |  |
| Change in other measure of body fat – stipulate type of measure (and give range/SD/SE) |  |  |  |
| Change in energy intake (add units) |  |  |  |
| Range/SD/SE* of change in energy intake at baseline |  |  |  |
