# Supplementary material for: Erratum to: systematic review and meta-analysis of the effect of increased vegetable and fruit consumption on body weight and energy intake
Source: BMC Public Health. 2017 Aug 17;17:662. doi: 10.1186/s12889-017-4664-2 (PMC5561641; doi:10.1186/s12889-017-4664-2)
Supplement: Supplementary file 1 — Derivation of standard errors for the difference in change in body weight between control and intervention from p-values (necessary for four studies included in the review). T-score and standard errors were imputed in Microsoft Excel, following the process outlined in the Cochrane Handbook (section 7.7.3.3) [8]. (DOCX 12 kb) [file 12889_2017_4664_MOESM1_ESM.docx]

Table S1: Derivation of standard errors for the difference in change in body weight between control and intervention from p-values (necessary for four studies included in the review)

|  | p-value | study size | difference | t-score | standard error |
| --- | --- | --- | --- | --- | --- |
| Smith-Warner 2000 | 0.57 | 185 | -0.09 | 0.57 | 0.16 |
| Weerts 2009 | 0.008 | 9 | -4.41 | 3.67 | 1.20 |
| Peterson 2011 | 0.08 | 176 | 0.4 | 1.76 | 0.23 |
| Christensen 2013 | 0.18 | 63 | -0.9 | 1.36 | 0.66 |

T-score and standard errors were imputed in Microsoft Excel, following the process outlined in the Cochrane Handbook (section 7.7.3.3)[8]
