# Supplementary material for: Erratum to: systematic review and meta-analysis of the effect of increased vegetable and fruit consumption on body weight and energy intake
Source: BMC Public Health. 2017 Aug 17;17:662. doi: 10.1186/s12889-017-4664-2 (PMC5561641; doi:10.1186/s12889-017-4664-2)
Supplement: Supplementary file 2 — Derivation of standard errors for the difference in change in body weight between control and intervention from standard errors for each group (necessary for three studies). *size of control group halved; and estimate of SE correspondingly adjusted (values used in calculations are shown in the table); the following formula used to calculate the standard errors for the difference in change in body weight between control and intervention was: ${SE}_{\mathit{\operatorname{int}}- con}=\sqrt{\frac{var_{int}}{n_{int}}+\frac{var_{con}}{n_{con}}}$SEint−con=varintnint+varconncon… (DOCX 12 kb) [file 12889_2017_4664_MOESM2_ESM.docx]

Table S2: Derivation of standard errors for the difference in change in body weight between control and intervention from standard errors for each group (necessary for three studies)

|  | Control Group | | Intervention Group | | Difference between groups |
| --- | --- | --- | --- | --- | --- |
|  | **n** | **Standard error** | **n** | **Standard error** | **Standard error** |
| Whybrow 2006 (Arm 1)* | 8 | 0.33 | 20 | 0.29 | 0.43 |
| Whybrow 2006 (Arm 2)* | 9 | 0.31 | 25 | 0.31 | 0.44 |
| Basu 2010 | 23 | 0.40 | 25 | 0.30 | 0.50 |
| Dow 2012 | 32 | 0.19 | 39 | 0.36 | 0.42 |

*size of control group halved; and estimate of SE correspondingly adjusted (values used in calculations are shown in the table); the following formula used to calculate the standard errors for the difference in change in body weight between control and intervention was: ${SE}_{int-con}=\sqrt{\frac{{var}_{int}}{n_{int}}+\frac{{var}_{con}}{n_{con}}}$…
